# A quantitative description of the spatial–temporal distribution and evolution pattern of world cultural heritage

**DOI:** 10.1186/s40494-021-00549-6

**Published:** 2021-07-05

**Authors:** Liang Yongqi, Yang Ruixia, Wang Pu, Yang Anlin, Chen Guolong

**Affiliations:** 1 Key Laboratory of Digital Earth Science, The Aerospace Information Research Institute, Chinese Academy of Sciences, Beijing, 100094 China; 2 University of Chinese Academy of Sciences, Beijing, 100049 China; 3 International Centre On Space Technologies for Natural and Cultural Heritage Under the Auspices of UNESCO, Beijing, 100094 China; 4 Engineering and Architecture, Beijing University of Civil, Beijing, 100044 China

**Keywords:** Cultural heritage, Quantification, Spatial–temporal distribution, Evolution pattern, Heritage policy, Heritage protection, Heritage classification system, Heritage discourses

## Abstract

Depicting the temporal and spatial evolution pattern of global world cultural heritage systematically and finely is the basis of heritage recognition and protection. In this study, 869 world cultural heritage inscriptions (through 2019) were selected as the research objects, and the times and types of each World Heritage site were manually annotated from more than 5000 pieces of data. Through time series modelling, the advantages of and changes in heritage declarations in different regions and periods were analysed, and the impact of heritage strategy on the number of heritage sites included in each region was evaluated. The results showed that the implementation of heritage policy greatly impacted each region, especially on the number of heritage sites in Asia and the Pacific region. Using the heritage era to carry out modelling analysis, from the perspective of the integrity of historical heritage cultural types, it is considered that there may be cultural heritage sites in the Caribbean and Latin America that have not been given enough attention. The modelling analysis results of era attributes can support the fairness of heritage determination. By calculating the frequency and peak value of heritage sites at the national scale, the frequency and peak value of each country in the top 10 list are used to characterize the ability of national declarations of cultural heritage and reveal the differences in the ability of each member country to declare heritage sites and the heritage era. By calculating the distribution density of the heritage era, this study finds that the world’s cultural heritage is not concentrated in the Middle Ages (600–1450) but the periods of Reformation and Exploration (1450–1700) and Progress and Empire (1850–1914). The above analysis shows that there are imbalances and strategic adjustment effects concerning regions, countries, eras and types in World Heritage list development. The composition types of heritage are complex, and the combination types have obvious changes in different periods. It is suggested that the strategy of world cultural heritage collection should be further optimized to fully guarantee the balance of regions, countries and types, and the heritage value should be fully considered in heritage protection with more diversity and complexity of types.

## Introduction

With the efforts of UNESCO, over the intervening 42 years from 1978 until 2019, the number of World Heritage sites has increased from the initial 12 to 1121. Of these, 869 are world cultural heritage sites, accounting for 77.5% of the total. At the same time, the number of states parties in the World Heritage Convention has increased from 20 to 167, which is the minimum number required at the beginning for entry and to enforce the international convention. World Heritage sites in China reached 55 in 2019, ranking it first in the world. This number includes 15 natural heritage sites (ranking first) and 37 cultural heritage sites (ranking fifth). As the number of sites has increased over time, many factors, such as World Heritage identification standards, policies, and contracting states' influence, have changed [1].

On the one hand, the World Heritage list can enhance the heritage sites' visibility and promote their protection. On the other hand, there are some problems, such as the difficulty of being completely fair in the heritage inscription process, leading to the neglect of some historical cultures that truly need attention and protection. In addition, the increase in both the number and types of heritage sites makes it difficult to protect heritage on a global scale [2]. At the same time, on the global scale, there are some imbalances in the brand effect of heritage [3], representative thoughts or discourses [4], and economic and policy support [5]. For the World Heritage list, these unbalanced factors are represented as the number and type of World Heritage differences between countries.

The quantitative description of these regional and type differences on the global scale and the analysis of the influencing factors and the implementation effect of heritage policies have important reference value for reducing future imbalances, increasing the attention given to neglected history and culture, and promoting heritage protection on a global scale. In the past, from the perspective of econometrics at the regional scale, the mechanism of these factors affecting the imbalance of heritage quantity has been given a more detailed explanation [6, 7]. However, there has been a lack of comprehensive research at the national scale.

World Heritage is an international confirmation of heritage, bringing significant political and economic benefits to the local area and the country. For example, heritage sites can become national landmarks and promote tourism development [8, 9]. Therefore, the World Heritage inscription is significantly affected by political factors and closely linked with its political influence and strategic interests [10]. Concerning a country's political influence, the most typical case is in authority vested by the World Heritage Committee (if the country holds a seat) [11]. Furthermore, World Heritage is intertwined in the ecology of exchanging gifts and mutual benefits between political alliances [12]. Existing studies only focus on case studies on imbalances concerning national heritage and need to be further recognized [13] to improve the fairness of World Heritage inscriptions.

The imbalance of World Heritage sites is reflected not only in number but also in cultural connotation. Historic towns, religious relics and some periods of "elitist" (rather than local) buildings fill the list, leading to a serious tilt in the discourse of heritage representatives [14]. Therefore, the diversity of heritage types needs to be further strengthened [15]. To prevent world cultural heritage inscriptions from becoming a global obliteration and exclusion rooted in religion and nationalism [16], heritage needs to be humanized and localized instead of focusing on Western ideas of expanding into colonies and marginalizing indigenous cultures, resulting in the concealment and loss of culture [17].

Given the problems existing in the inscription of the World Heritage site, in 1994 the World Heritage Committee launched a global strategy to make World Heritage balanced, feasible and representative (hereinafter referred to as the “Global Strategy”) [18]. Previous studies have shown that the strategy has not achieved the desired effect and cannot achieve the balanced distribution of heritage sites [19].

An increasing number of studies have shown that unbalanced objects need to fall on the national scale [20–22]. To ensure the diversity of heritage, higher requirements have been put forward regarding heritage property characteristics [23]. Simultaneously, the effects of heritage policy implementation need to be compared with heritage characteristics in time series [24–26]. Therefore, based on the inscription time (1978–2019) and heritage era (4,000,000 BCE–1957 CE), this study analyses the quantitative distribution and evolution process of world cultural heritage in regions and countries from the perspective of regional, national and type in time and space dimensions. With the influence coefficient index, frequency and peak value, overlapping degree of heritage types, and heritage density we quantitatively depict the imbalance of world cultural heritage, reveal the characteristics of spatial–temporal distribution and influence mechanism of World Heritage, and provide a reference for policy adjustment, inscription and protection of cultural heritage.

## Data and methods

### Data source and annotation

The research data are extracted from the World Heritage list on the UNESCO-WHC official website, and more than 4000 heritage introduction pages were manually retrieved. The World Heritage list contains information on the name, type, country, and region of World Heritage Sites through December 2019, as well as criteria and inscription time. There are three types of heritage sites on the list: natural heritage, cultural heritage and mixed heritage. In this study, World Heritage cultural sites were selected as the research object, with 869 sites. For the cultural heritage type and era attribute data, the information pages containing cultural heritage attributes were retrieved one by one and were then manually extracted and labelled. A table was then formed for the analysis.

#### World cultural heritage type and annotation

For the types of world cultural heritage, the classification systems will vary with the classification basis. This research mainly referred to the classification system of Asian heritage protection practice, namely, six basic types: monument and historic buildings, historic urban, archaeological sites, cultural landscape, industrial heritage, and underwater heritage [27, 28] (Table 1). In type annotation, for cultural heritage with a special composition form, the node features were used to classify them into each corresponding type [29]. Some cultural heritage sites contain multiple attributes and cannot be simply classified into a single type [30]. During data processing, all the basic types of cultural heritage were marked. Based on the above data processing ideas, each cultural heritage site's type or combination type was obtained, and a table with 21 types or combination types was formed (Table 2).

**Table 1:** Definitions of the six basic types of world cultural heritage [26, 27]

Type	Definition
Monument and Historic Buildings	This category encompasses individual, built heritage resources and architectural complexes and their settings. These are deemed to possess heritage significance and have been or will be listed or declared for protection and conservation
Historic Urban	A historic urban site or heritage group comprises several related and spatially adjacent, or at least proximate, resources, all of which are of heritage value individually or contribute to the group's overall heritage significance
Industrial Heritage	Industrial heritage refers to the physical remains of the history of technology and industry, such as manufacturing and mining sites, as well as power and transportation infrastructure
Archaeological Sites	An archaeological site comprises any combination of structural remains, artefacts and ecological elements within a culturally modified soil matrix. A site may lie entirely beneath the surface or appear partially above it. It may be fully or partially excavated, or it may be known only through textual reference or subsoil or remote sensing
Cultural Landscape	A cultural landscape is a geographic area, including both cultural and natural resources and the wildlife or domestic animals therein, associated with a historic event, activity, person or exhibiting other cultural or aesthetic values
Underwater Heritage	Underwater cultural heritage means all traces of human existence having a cultural, historical or archaeological character which have been partially or totally underwater, periodically or continuously, for at least 100 years

The definition of basic types is summarized according to the relevant contents in the papers [27, 28]

**Table 2:** Statistical results of world cultural heritage combination types

Code	Basic type 1—Basic type 2—Basic type 3—Basic type 4	Count
1	Monuments and Historic Buildings	270
2	Historic Urban—Monuments and Historic Buildings	233
3	Archaeological Site	13
4	Archaeological Site—Monuments and Historic Buildings	68
5	Archaeological Site—Historic Urban—Monuments and Historic Buildings	150
	Count of Archaeological Site	231
6	Under Water Site—Monuments and Historic Buildings	1
7	Under Water Site—Archaeological Site—Historic Urban—Monuments and Historic Buildings	1
	Count of Under Water Site	2
8	Industrial Heritage—Monuments and Historic Buildings	6
9	Industrial Heritage—Historic Urban—Monuments and Historic Buildings	16
10	Industrial Heritage—Archaeological Site—Monuments and Historic Buildings	1
11	Industrial Heritage—Archaeological Site—Historic Urban—Monuments and Historic Buildings	1
	Count of Industrial Heritage	24
12	Cultural Landscape	7
13	Cultural Landscape—Monuments and Historic Buildings	44
14	Cultural Landscape—Historic Urban	2
15	Cultural Landscape—Archaeological Site	5
16	Cultural Landscape—Industrial Heritage	1
17	Cultural Landscape—Historic Urban—Monuments and Historic Buildings	20
18	Cultural Landscape—Archaeological Site—Monuments and Historic Buildings	15
19	Cultural Landscape—Industrial Heritage—Monuments and Historic Buildings	1
20	Cultural Landscape—Archaeological Site—Historic Urban—Monuments and Historic Buildings	9
21	Cultural Landscape—Industrial Heritage—Historic Urban—Monuments and Historic Buildings	5
	Count of Cultural Landscape	109
	All Count	869

#### World cultural heritage era and annotation

**Fig. 1:**
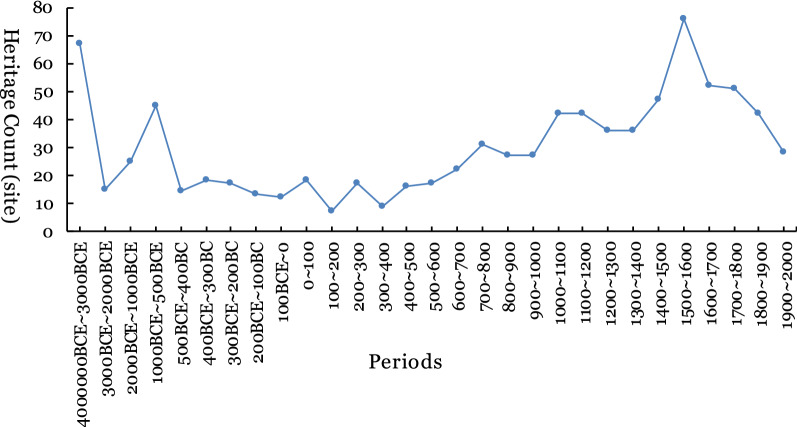
Era attributes of world cultural heritage

World cultural heritage is the material that remains from human history’s development and has distinct characteristics over time. Heritage eras were defined and extracted according to the following principles: (1) The cultural heritage of single buildings, such as churches, bridges, and canals, have a simple and clear era; thus, we took this construction era as a heritage age. (2) The architectural complex and industrial heritage are periods lasting for a long time, and the construction time of the earliest building was chosen as the heritage era. (3) Historical town heritage areas are areas that experienced the complex process of establishment, expansion and reconstruction. If there is a typical architectural era mark in the documentation, the era was selected. If there is no sign, the age of the town was selected. (4) The archaeological site's era is referred to the earliest age in the archaeological excavation reports. (5) Cultural landscape heritage is often related to the formation time of the local landscape that continues to grow and experience organic evolution; hence we choose the era of landscape formation as the heritage era. (6) Because a certain cultural heritage may have complex combination types, the earliest era among the basic types was selected as the heritage era. According to the above principles, we formed the heritage age data table. The heritage era's time span is from 4,000,000 BCE to 1957 CE (Fig. 1), and the data accuracy supports period division (Fig. 1).

With the support of fine heritage era attribute information, referring to the Smithsonian Institution's division rules for known human history [31, 32], the heritage era is simplified into eight periods, corresponding to different cultural periods (Table 3): Human Origin (8 MYA-3000BCE), Early Civilization (3000-700BCE), the Classic Age (700BCE-600CE), Trade and Invention (600–1450), The Reformation and Exploration (1450–1700, which corresponds to the era of great navigation and colonialism originating in Europe), Revolution and Industry (1700–1850), Progress and Empire (1850–1914, corresponding to the era of imperialism in Europe), and Technology and Superpowers (1914–2011).

**Table 3:** Cultural period and time period

Code	Cultural period	Start and end time
1	Human Origin	8 MYA–3000BCE
2	Early Civilizations	3000–700BCE
3	The Classic Age	700BCE–600CE
4	Trade and Invention	600–1450
5	Reformation and Exploration	1450–1700
6	Revolution and Industry	1700–1850
7	Progress and Empire	1850–1914
8	Technology and Superpowers	1914–2011

### Quantitative characterization of the spatial–temporal characteristics of world cultural heritage

To fully reveal the difference and imbalance of the number of heritage sites between regions or countries, this study evaluates the influence of regions and the historical integrity of heritage by modelling the number of regional heritage sites and evaluates the attention and advantage of world cultural heritage at the national scale and the historical richness of heritage by statistical frequency and peak value. By calculating the density of heritage and the overlap rate between basic types, the concentration degree of heritage quantity at different times and the richness of heritage types and their changes over time are described.

#### Time series modelling

Based on the number of new heritage sites (x) and the total number of heritage sites (y) globally in a certain period in each region, the impact coefficient of each region relative to the world is obtained by modelling in a time series. The formula is as follows:

1 $$\begin{array}{*{20}l} {y = {\text{a}}x + {\text{b}}} \hfill \\ {x \in \left\{ {x_{1} ,x_{2} ,...,x_{n} } \right\};y \in \left\{ {y_{1} ,y_{2} ,...,y_{n} } \right\}} \hfill \\ {SSR = \sum\limits_{{i = \{ 1,n\} }} {(\mathop {y_{i} }\limits^{ \wedge } - \overline{y} )^{2} } ;SST = \sum\limits_{{i = \{ 1,n\} }} {(y_{i} - \overline{y} )^{2} } } \hfill \\ {R^{2} = \frac{{SSR}}{{SST}}} \hfill \\ \end{array}$$

In the formula, ‘a’ and ‘b’ are the regression coefficient and constant, respectively. The identifier *x*_*i*_ refers to the number of heritage sites in a certain region at a certain time point or period; *y*_*i*_ refers to the total number of new heritage sites in the world at a certain time point or period; SSR is the sum of regression squares; and SST is the sum of total squares. When the time variable is the inscription time, *i* corresponds to the year. For example, as the span 1978–2019 contains 42 years, *i* ranges from 1 to 42. When the time variable is the heritage era, *i* corresponds to the heritage era, such as the 8 periods (Table 3); thus, *i* ranges from 1 to 8.

When the time variable is the inscription year of heritage, the influence coefficient R^2^ represents the region's influence on the number of world cultural heritage sites in this period, which is defined as the "heritage represent degree". When the heritage era is selected as the time variable, the influence coefficient R^2^ can reflect the historical integrity represented by the WC of each region, which is defined as the "heritage integrity degree".

#### Frequency and peak value statistics

According to the statistics on the annual number of newly added heritage sites in each country, the top 10 countries and their newly added numbers were obtained for the 42 years. The frequency and peak value of the top 10 countries in all years were further counted, which was used as the evaluation index for the superiority of heritage declaration at the national level. The number of heritage sites distributed by countries in each era was counted to obtain the top 10 countries and their new numbers. The frequencies and peak values of the top 10 countries were further counted as the national scale heritage historical and cultural richness evaluation index. The formula is as follows:

2 $$\begin{array}{*{20}l} {\text{Count}_{{\text{top}\_10(m)}} {\text{ }} = {\text{ Max}}\_10(\{ \text{Count}_{{m1}} , \ldots ,\text{Count}_{{mn}} \} )} \hfill \\ {\text{Frequence}_{t} {\text{ }} = {\text{ Len}}(\{ \text{Count}_{{\text{top}\_10(m1)}} ,\ldots,\text{Count}_{{\text{top}\_10(mn)}} \} _{{if(n \in t)}} ){\text{ }}} \hfill \\ {\text{Max}_{t} {\text{ }} = {\text{ }}\text{Max}(\{ \text{Count}_{{\text{top}\_10(m1)}} ,\ldots,\text{Count}_{{\text{top}\_10(mn)}} \} _{{if(n \in t)}} ){\text{ }}} \hfill \\ {n \in \{ \text{Country}\_\text{code}\} _{m} ;t \in \{ \text{Country}\_\text{code}\} _{{\text{top}\_10}} } \hfill \\ {m \in \{ \text{Year}\_\text{code}\} \text{or}\{ \text{Period}\_\text{code}\} } \hfill \\ \end{array}$$

where Count_top_ 10_ (*m*) is the number of the top 10 heritage sites in year *m* (or the *m*-th era) and the corresponding national collection; frequency is the frequency of the top 10 countries in all years (or all eras); Max_*t*_ is the peak of the number of heritage sites in 42 years (or all eras) of the top 10 countries; *n* is the country of heritage included (or the *m*-th era) in year *m*; *m* is the inscription year of heritage (e.g., 1–42) or heritage era (e.g., 1–8, Table 3); and *t* is for countries that appear in the top 10 of the rankings by year or era.

#### Heritage density

In this study, the ratio of the number of world cultural heritage sites to the length of time in a certain era is defined as the density of cultural heritage to describe the concentration degree of the distribution quantity of cultural heritage in each era. The formula is as follows:

3 $$Heritage_{{den}} = \frac{{Count_{{her}} }}{{Range_{{per}} }}$$

where Count is the number of cultural heritage sites distributed in each era, Range is the length of time of the era, such as 500 years, and ‘her’, ‘per’, and ‘den’ represent the ‘heritage’, ‘period’ and ‘density’, respectively.

#### Type overlap rate

In this study, six basic types of cultural heritage were taken as references (Table 1), and the overlap rate between each basic type and other types was calculated to describe the complexity and multitype characteristics of cultural heritage. The formula is as follows:

4 $$Rate_{{OverlapBinA}} = \frac{{Count_{{A\_B}} }}{{Count_{A} }}$$

where Count_A_ is the number of heritage sites of a basic type, such as type A; Count_A_ B_ is the number of heritage sites with basic types A and B; and A is not the same as B.

## Results and discussion

### Spatial–temporal evolution of world cultural heritage numbers and types

#### Change of heritage quantity over time

**Fig. 2:**
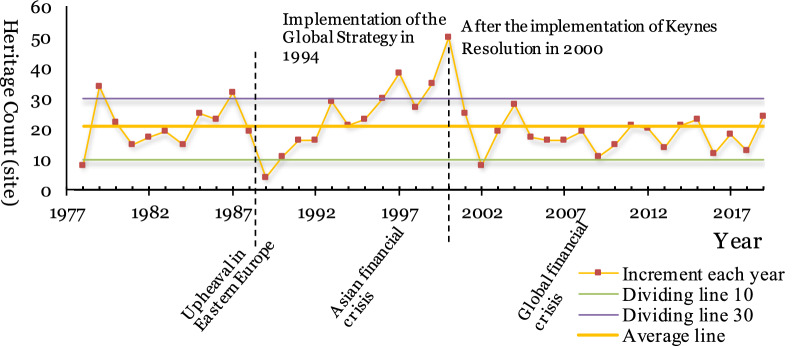
The change in the annual increments of world cultural heritage sites over time, corresponding to major events

From the change in the annual increment of world cultural heritage sites over time (Fig. 2), the count has the characteristics of a phased distribution, and previous studies have similar conclusions [1]. In 42 years, approximately 3/4 of the years, world cultural heritage sites' annual increase was between 10 and 30. The number of new cultural heritage sites increased significantly less in 1989 and 2002, and the peak number appeared in 1997 and 2000.

There is an obvious corresponding relationship between the number of new inscription heritage sites and global or regional political, economic or policy events over time. For example, the drastic changes in Eastern Europe in approximately 1988 and the restructuring of the global order corresponded to the low annual increment in 1989. Additionally, the promulgation of the “Keynes Resolution” in 2000 resulted in a significant decrease in the annual increment from 2000 to 2002 and the subsequent microwave dynamic development stage. The "No more than one heritage declaration per year by the Contracting Parties with existing projects" regulation in the Keynes Resolution and the "Up to two World Heritage sites to be declared by the Contracting Parties each year" in the amendment to the Keynes Resolution in 2004 have played important roles.

On the whole (Fig. 2), global political and policy factors have had great and long-term impacts on the annual increment of new heritage sites. When economic factors fluctuate, the impact on the annual increment of heritage is small and short-term.

#### Spatial–temporal distribution of world cultural heritage at regional and national scales

The World Heritage list is divided into five regions: Asia and the Pacific, Europe and North America, Africa, Latin America and the Caribbean, and Arab regions. Among them, the Arab region is listed separately. Europe and North America are merged, and Latin America and the Caribbean are merged into one region, reflecting the consistency of regional cultural identity, political alliances, and economic development.

**Fig. 3:**
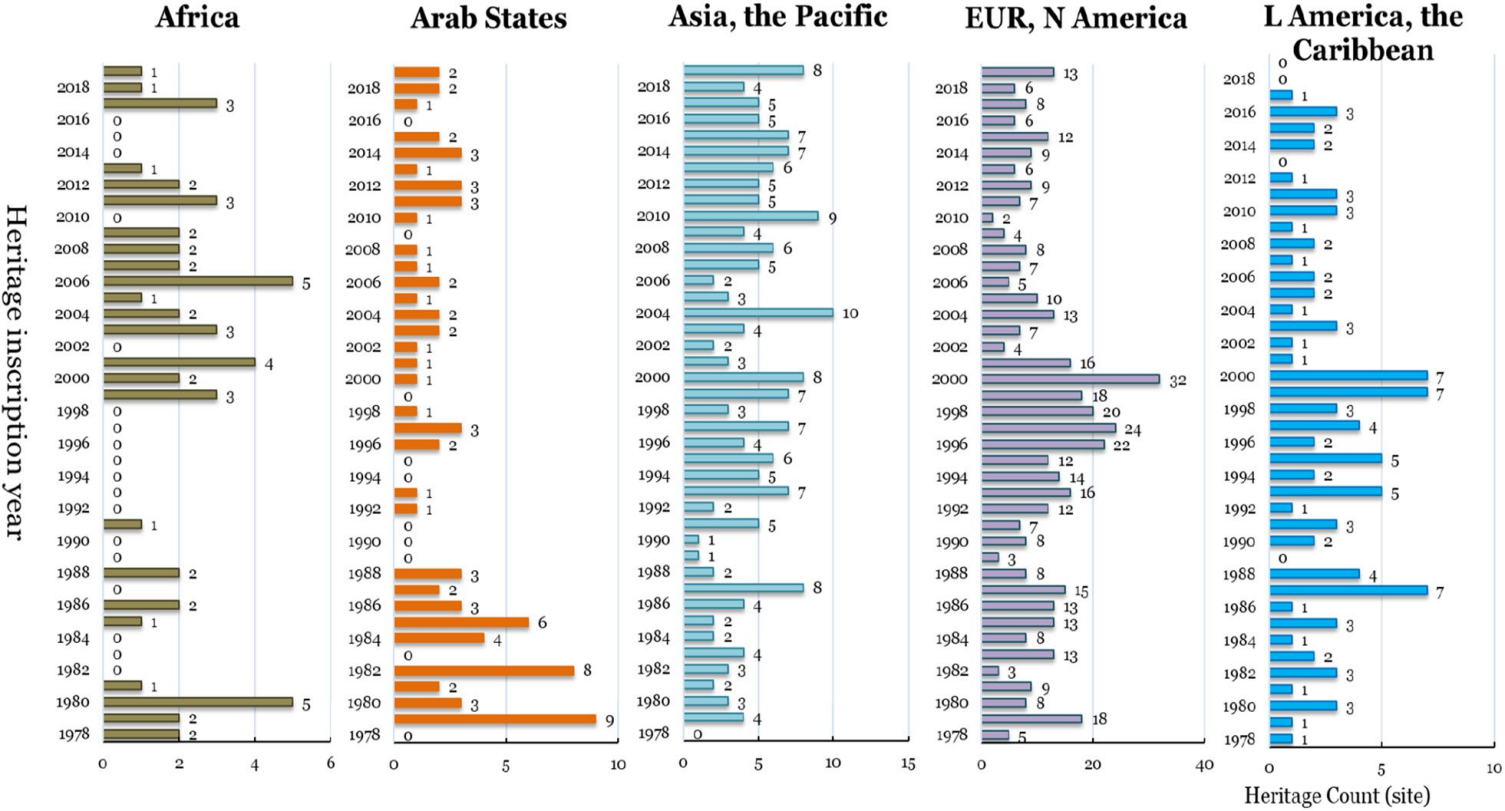
Changes in annual increment number of world cultural heritage sites in different regions over time

According to the distribution of the annual increment of WC in different regions (Fig. 3), there are 18 and 10 years without any increases in the African and the Arab regions, respectively. There were increases concentrated in 1992–2001 in Europe and North America, Latin America and the Caribbean, and the Asia and the Pacific region. Newly added amounts are concentrated after 2003, accounting for half of the total number of regions. The imbalance of the number of newly added heritage sites among the above regions over time reflects the differences in the degree of attention given by the different regions to world cultural heritage and the regional influence of World Heritage policies.

**Fig. 4:**
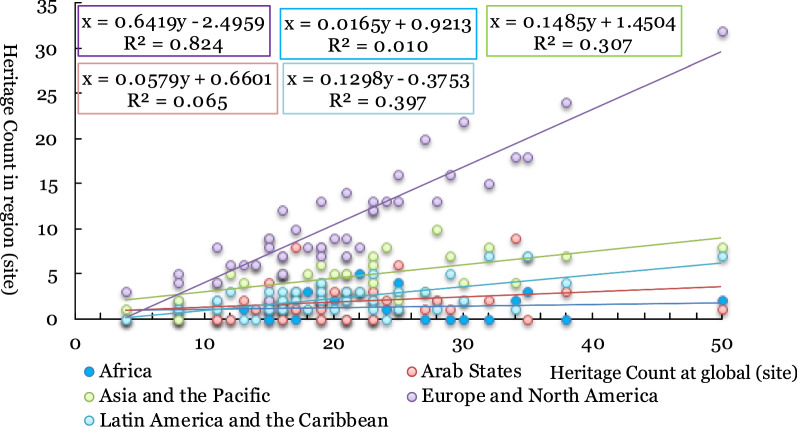
The relationship between the annual increment of world cultural heritage sites in different regions and the total number of world cultural heritage sites in that year (1978–2019)

From the impact coefficient of the annual increment of world cultural heritage sites in each region from 1978 to 2019 and the total number of new world cultural heritage sites in that year (Fig. 4), Europe and North America are the dominant regions with the highest heritage representation degree (R^2^ is 0.824). The calculation results of other regions' influence coefficients are not significant (R^2^ is less than 0.5), indicating that the heritage representation degree of other regions is relatively weak.

To further analyse the impact of heritage policy on the annual number of new heritage sites, the period model of heritage policy impact is selected to compare and analyse the changes in the influence of different regions before and after the implementation of heritage policy to reflect the effect of heritage policy implementation on each region. Concerning the regional impact of the Global Strategy (proposed in 1994) and the Keynes Resolution (promulgated in 2000), three periods are selected to calculate the regional impact coefficients: 1978–1994 (17 years), 1978–2000 (23 years) and 1978–2019 (42 years).

From the impact coefficient of each region in the three periods mentioned above, the implementation of the Global Strategy in 1994 did not change world cultural heritage status in Europe and North America, which was the global heritage issue of most concern. After implementing the Global Strategy, the heritage display degree of Asia and the Pacific Region increased slightly, and R^2^ increased from 0.564 to 0.653. After implementing the Keynes Resolution, Asia and the Pacific region showed a slight decrease in R^2^ in the Pacific region, decreasing from 0.653 (weakly significant) before the implementation of the Keynes Resolution, to 0.307 (insignificant). The resolution significantly weakened heritage visibility in Asia and the Pacific but had little impact on Europe and North America (Table 4).

**Table 4:** The influence coefficient of the world cultural heritage site numbers in different regions and the world during three periods

	Africa	Arab States	Asia and the Pacific	Europe and North America	Latin America and the Caribbean
Period 1 (1978–1994)	0.025	0.237	0.564	0.707	0.336
Period 2 (1978–2000)	0.034	0.033	0.653	0.838	0.491
Period 3 (1978–2019)	0.011	0.065	0.307	0.824	0.397

From the perspective of the change in heritage before and after the implementation of the policy (Table 4), the implementation of the two policies is more favourable for Europe and North America and Latin America and the Caribbean in terms of world cultural heritage declarations, but negatively impacts Asia and the Pacific.

**Fig. 5:**
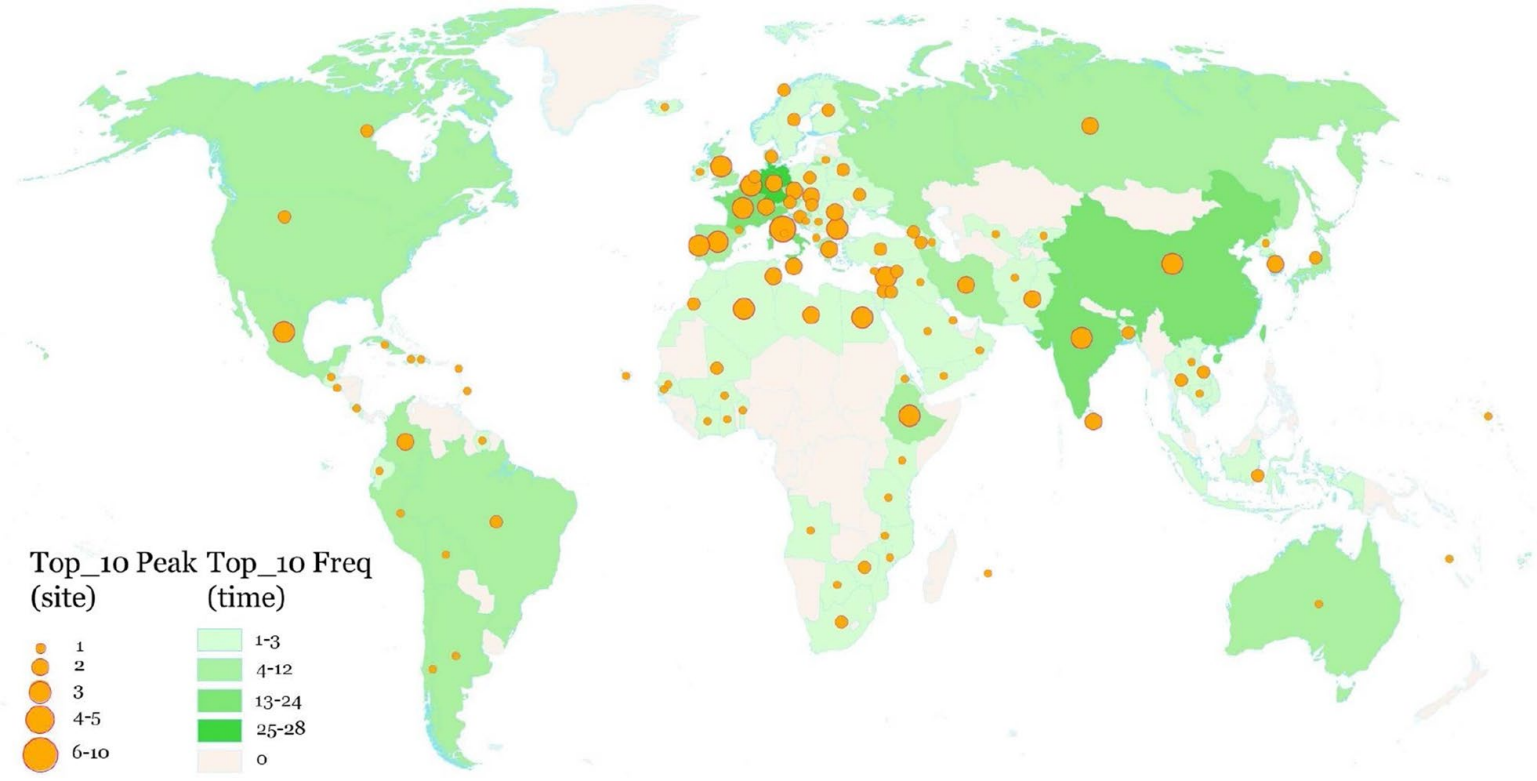
Frequencies and the peak values of countries in the top 10 list of world cultural heritage sites annual increment numbers from 1978 to 2019

According to the statistics of the frequency and peak value of the number of newly added heritage sites in each country from 1978 to 2019 (42 years), it can be seen that the number of newly added heritage sites in the top 10 of the same year appears. The countries with high superiority in world cultural heritage declarations can be divided into three tiers: Germany ranked first with 28 instances; the second tier was France, Italy, Spain, India and China; and the third tier was Japan, Iran, Brazil and Mexico. The countries in most of Africa, the Arabian Peninsula, Central Asia, Southeast Asia, Eastern Europe and Northern Europe had low superiority in world cultural heritage declarations (Fig. 5).

From the perspective of the peak number of world cultural heritage sites added over the years (Fig. 5), the countries with high peak values are concentrated in Europe and the Mediterranean. There are also large differences in the superiority of world cultural heritage declarations among countries in the region. For example, the highest peak value was in Italy, which had 10, while many Eastern European countries had only one peak value.

#### Changes of world cultural heritage types over time

**Fig. 6:**
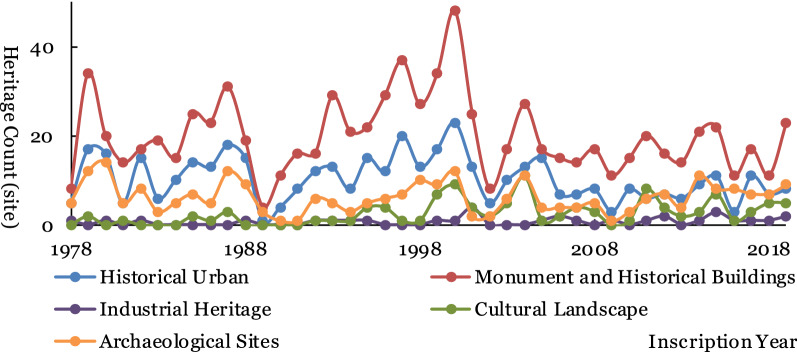
Statistics of basic types of world cultural heritage annual increment numbers from 1978 to 2019

According to the types of new world cultural heritage sites from 1978 to 2019 (Fig. 6), the number of monument and historic buildings is the largest, followed by historic urban. The number of historic urban, monument and historic buildings, and archaeological sites has the same trend over time. The number of cultural landscape heritage types was small before 1993 and then fluctuated because the concept of cultural landscape was included in other basic types of world cultural heritage before 1994.

### Characteristics of the times of world cultural heritage

#### Quantitative distribution of heritage in different periods

**Fig. 7:**
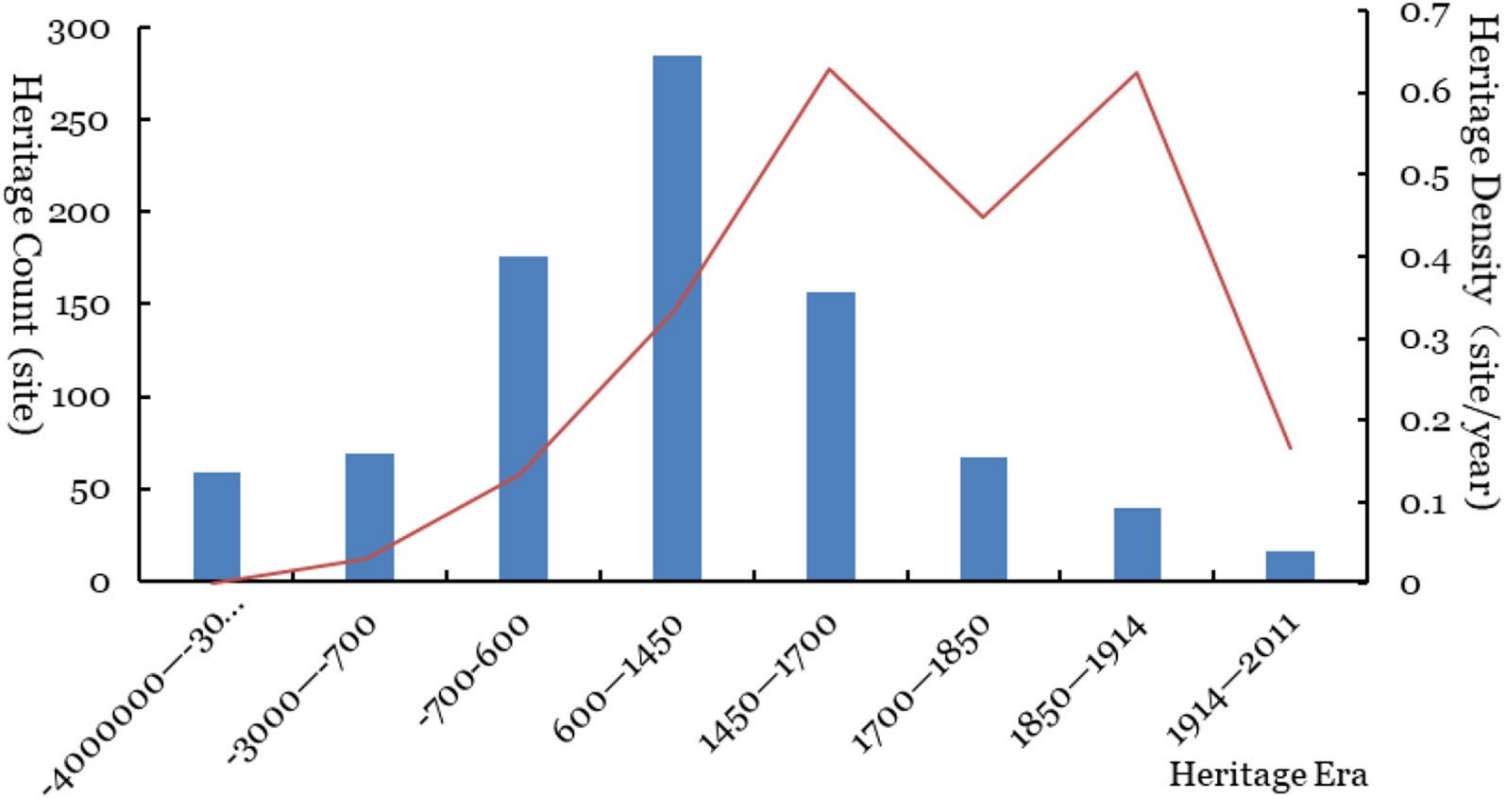
Distribution of world cultural heritage counts in different eras

Surveying the distribution of world cultural heritage sites in different periods (Fig. 7), the Trade and Invention (600–1450, i.e., the middle ages of Europe) era has the largest, followed by the Classic Age (700 BCE-600 CE) and The Reformation and Exploration (1450–1700). From the perspective of world cultural heritage density, heritage sites are concentrated in The Reformation and Exploration (1450–1700) and Progress and Empire (1850–1914) periods, followed by Revolution and Industry (1700–1850). Although the largest number of heritage sites are in the Trade and Invention (600–1450), the degree of concentrated distribution of heritage sites was not obvious.

Combined with each period's cultural background, the battles of various forces in medieval Europe (600–1450) left a large number of castles and military buildings, corresponding to the largest number of world cultural heritage sites distributed in that era. The Roman Empire's expedition and rule in the Classic Age (700BCE–600CE) created many ancient cities and sites, with a corresponding number of world cultural heritage sites distributed in that era. During the Revolution and Industry (1700–1850) period, as European powers grew stronger and began exploring, they plundered resources and expanded their territory on a global scale through colonialism and imperialism; this resulted in 14 sites. Culture and economy collided fiercely worldwide, and the corresponding world cultural heritage sites are significantly concentrated in the above two periods. During Revolution and Industry (1700–1850), colonial and local revolutionary gunpowder erupted, and political turmoil caused cultural changes. The degree of concentrated distribution of heritage sites decreased, forming a local trough.

#### Heritage era characteristics at regional and national scales

**Fig. 8:**
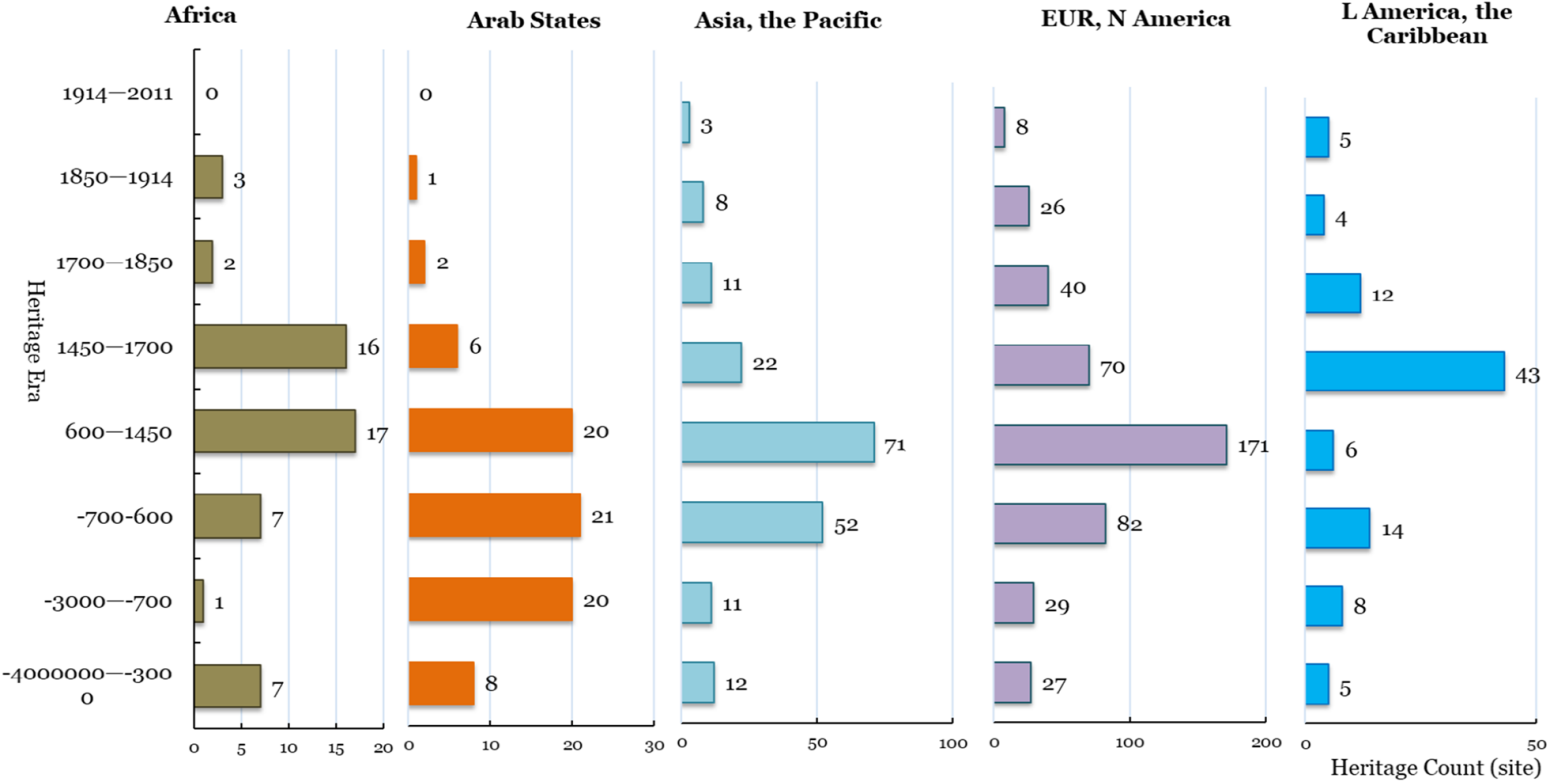
Regional differences in the era distribution of world cultural heritage sites

The world cultural heritage sites of Asia and the Pacific region and Europe and North America are concentrated in the Trade and Invention (600–1450) period. Latin America and the Caribbean heritage sites are concentrated in The Reformation and Exploration (1450–1700), which is mostly colonial. The heritage sites of the Arab region are mainly distributed in Early Civilization (3000–700 BCE), the Classic Age (700 BCE-600 CE), and Trade and Invention (600–1450) (Fig. 8).

**Fig. 9:**
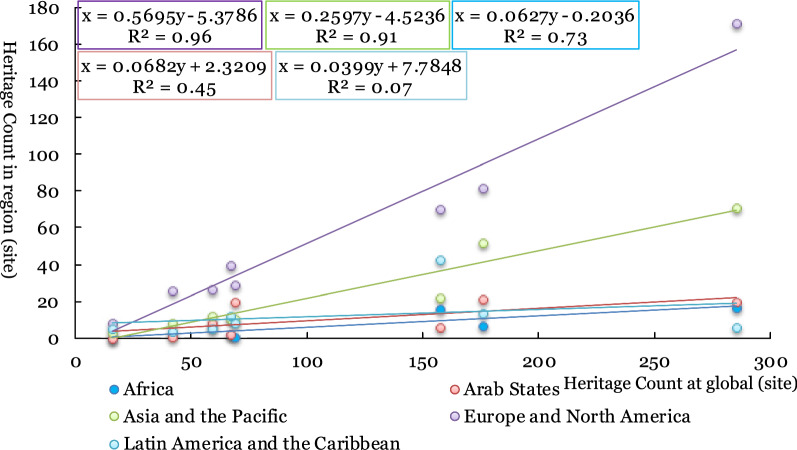
The relationship between the number of world cultural heritage sites in different regions and the total number of world cultural heritage sites in the era

From the influence coefficient of the distribution of world cultural heritage sites in different regions and the total number of global world cultural heritage sites in the 8 periods (Figs. 8, 9), the cultural heritage integrity of Europe and North America is the highest (R^2^ is as high as 0.963), and the cultural heritage integrity in Asia and the Pacific is second (R^2^ is 0.917). Combined with the number and representation of world cultural heritage sites in Asia and the Pacific (Fig. 3 and Table 3), the heritage policy is unfavourable to Asia and the Pacific region. The number of world cultural heritage sites in the region is less than half of Europe and North America (190/453). However, the world cultural heritage sites in the region can still maintain fine historical integrity. The world cultural heritage sites in the Arab region and Latin America and the Caribbean lacks some culture. From the distribution of world cultural heritage sites in each era, the historical and cultural areas to be concerned about in the Arab region are in The Reformation and Exploration and after 1450 for Latin America and the Caribbean. The historical history and culture to be concerned about are all eras except for the Reformation and Exploration (before 1450 and after 1700).

**Fig. 10:**
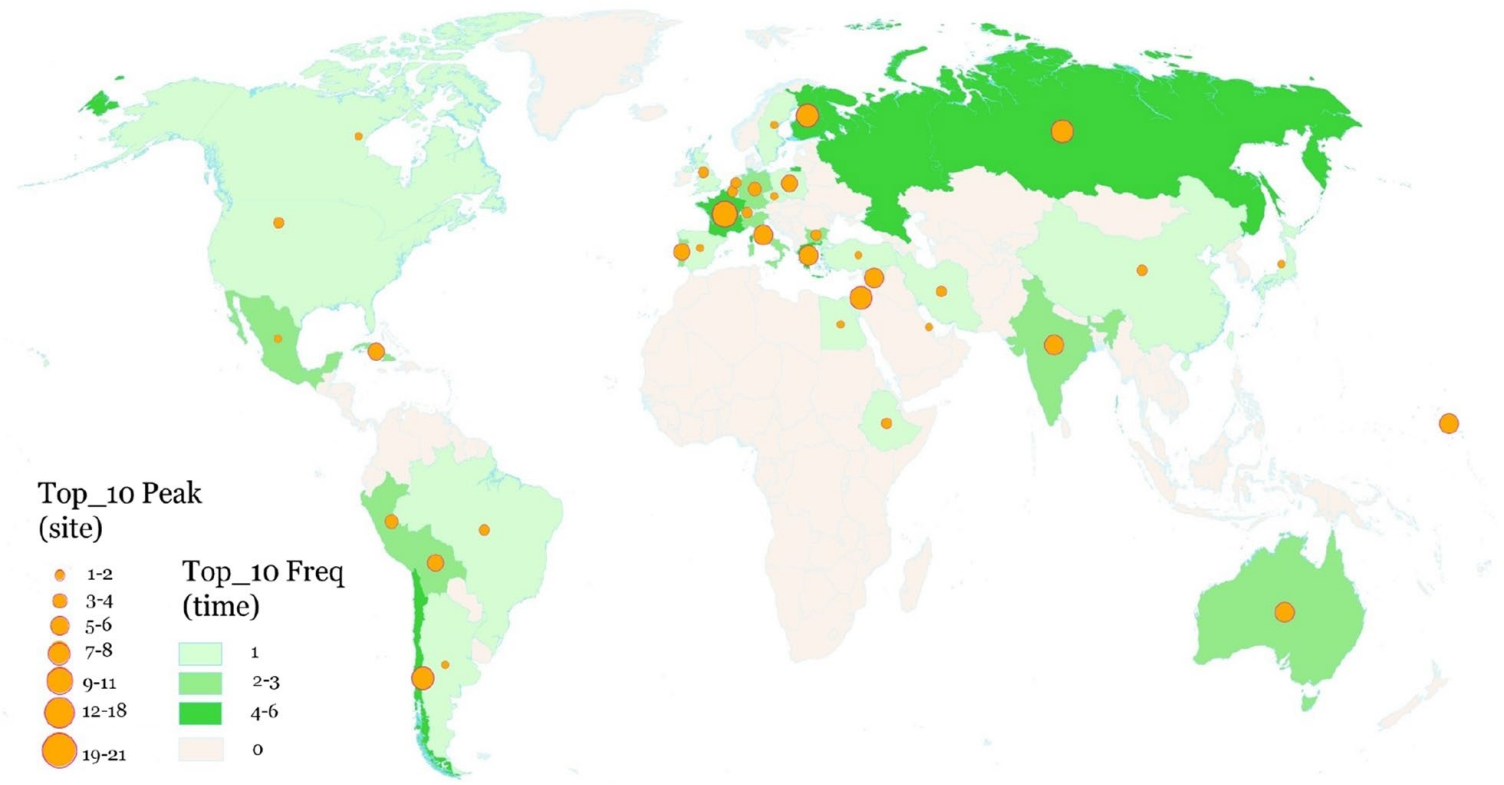
Frequencies and peaks of countries in the top 10 list of world cultural heritage sites, showing distribution numbers for the 8 eras

According to the frequency and peak value of the top 10 world cultural heritage site distributions at different times (Fig. 10), the countries with high historical richness can be divided into two groups. The first echelon is France, Finland, Russia, Argentina, Greece, with the highest historical richness, encompassing more than four generations. The second-tier countries are Mexico, Peru, Bolivia, India and Australia. In China, Iran, Turkey, Egypt, Ethiopia, Spain, Poland and other countries, the distribution of heritage sites is poor; only in individual periods are world cultural heritage site distribution numbers dominant. The countries with low historical richness are mainly distributed in Africa, the Arabian Peninsula, and East South Asia. South Asia, Central America, Eastern Europe and Central Asia have never appeared in the top 10 list. There are more than 12 world cultural heritage sites in France, Russia, Finland, Greece, Italy, Argentina, India and Australia.

#### Evolution of heritage types with time

**Fig. 11:**
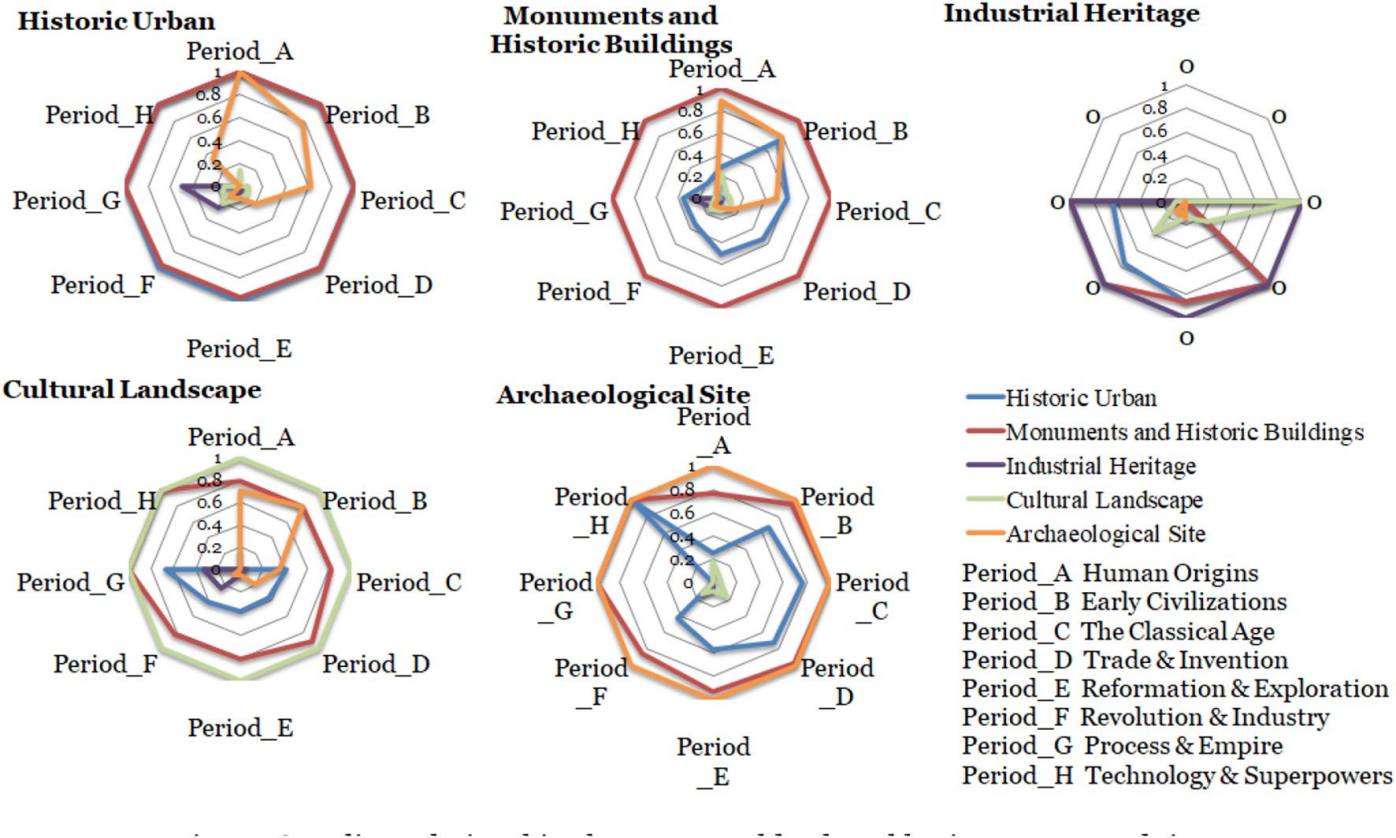
Coupling relationships between world cultural heritage types and times

World cultural heritage often includes many types, and there will be different types of combinations in different historical periods. By analysing the coupling relationship between WC's types and eras (Fig. 11), we can see the overlapping (inclusive) relationship between the main heritage types and other types in different periods and reveal the time evolution characteristics in the combination of heritage types. For example, more than 90% of the world cultural heritage sites, whose main body comprises historical towns, also contains the types of archaeological sites and historic buildings in the whole period. The historical towns in the Human Origin period (8 MYA–3000BCE) also belong to archaeological sites, and the two types completely overlap. However, over time, archaeological sites in historical towns are gradually decreasing. Until the Reformation and Exploration (1450–1700) era, only 10% of the historical towns contain archaeological sites. The overlapping rate of the main types of monuments and ancient buildings with archaeological sites and historical towns shows a similar trend of decreasing over time, in which the overlapping rate of the main types of historical towns begins from the Early Civilization (3000–700BCE) to The Reformation and Exploration period (1450–1700). From there, the overlapping rate decreases steadily. Industrial heritage first appears in the Classic Age (700BCE–600CE); the latest period is Progress and Empire (1850–1914). Between the Reformation and Exploration (1450–1700), the overlap rate with historic sites, ancient buildings and historical towns was more than 90%. In the Revolution and Industry period (1700–1850), the overlap rate with historical towns decreased. Over the whole era, the overlap rate between cultural landscapes and archaeological sites or historic buildings decreased. The overlapping rate of archaeological sites is approximately 80% in the Human Origin period (8 MYA-3000BCE) and Early Civilization (3000–700 BCE). The overlapping rate of archaeological sites and historic sites or ancient buildings is approximately 80% in all periods. The overlapping rate of historical towns is approximately 80% in Early Civilization (3000–700 BCE). The Classic Age (700 BCE-600 CE), Trade and Invention (600–1450), and The Reformation and Exploration (1450–1700) were all approximately 60%. From the above analysis, we can see that the same cultural heritage may present different value types in different periods, reflecting the complexity of cultural heritage types. At the same time, it is necessary to recognize and protect all types of value in heritage protection.

## Conclusions

Based on UNESCO's world cultural heritage List, this paper quantitatively describes the temporal and spatial evolution process of the number and type of heritage in terms of regional and national scales, combined with manually retrieved attribute data such as heritage type and era. The main conclusions are as follows:

1. The annual increase in the number of WC Heritage Sites is affected by global politics and policies, economic crises and other factors. Among them, World Heritage policies comprise an important factor affecting the number of new cultural heritage sites. The Global Strategy and Keynes Resolution have not changed the dominant position of Europe and North America in heritage quantity. After the Global Strategy was put forward in 1994, Asia and the Pacific region’s heritage visibility received a brief and tiny increase. However, after the Keynes Resolution promulgation in 2000, the region's heritage visibility decreased sharply. This is because Keynes Resolution limit the number of nominations per year on a national basis. So, we hope that the new heritage policy can refer to the historical and cultural connotation of each country, improve the limitation rules, and give countries with a long history and culture more quotas for declaration.
2. The statistics on the frequency and peak value of the newly added cultural heritage sites in the top 10 countries reflect each country's participation and advantage in the declaration of heritage sites, which has a certain significance for balancing the number of selected heritage sites among countries in the future.
3. World cultural heritage embodies the elements of colonialism and imperialism. The indigenous cultures in Latin America, the Caribbean and Africa need more attention. It is not only necessary to ensure the quantitative balance of the world cultural heritage in these areas, but also to thoroughly evaluate the historical and cultural connotation of the existing heritage, avoid the historical and cultural deviation represented by the listed heritage, and truly protect the common heritage of mankind, rather than some cultural heritage.
4. Compared with Europe and North America, the number of cultural heritage sites in Asia and the Pacific is much lower. Still, the world cultural heritage sites in this region shows the integrity of regional cultural sequence times. This provides an optimized plan for the nomination of heritage for those countries that may not be able to equalize the number of heritages under the constraints of existing heritage policies, that is, priority is given to nomination of heritage that represents the country’s lack history and culture.
5. There are many kinds of overlaps among the basic types of cultural heritage. The overlapping rate for each type of heritage has obvious differences in the distribution of times, which can reference the protection of cultural heritage sites on a global scale.

Compared with previous studies, this study comprehensively details the research scale for each country, supplements the fine-type and era attribute information of cultural heritage sites, evaluates the imbalance of world cultural heritage sites in quantity and connotation with more quantitative indicators, reveals its spatial and temporal evolution pattern, and analyses the impact differences of heritage policies on different regions. It is an important reference for the formulation and evaluation of property policy and the declaration and protection of cultural heritage. However, there are also some shortcomings in this paper, such as world cultural heritage being a dynamic process. Due to the complexity of data annotation, this paper only uses the initial construction or formation time to represent the heritage era, which is relatively rough. Follow-up research will extract the time and event information of heritage process nodes from the collected text data and describe heritage's dynamic process more carefully to reflect the rich historical culture.

## Data Availability

The data sets used or analysed during the current study are available from Liang Yongqi upon reasonable request, and his email address is yongqeeleeao@foxmail.com.
